# Subcutaneous panniculitis‐like T‐cell lymphoma post‐mRNA‐1273 COVID‐19 vaccination

**DOI:** 10.1002/ccr3.7143

**Published:** 2023-04-05

**Authors:** Sho Ukishima, Taiju Miyagami, Mari Arikawa, Seiko Kushiro, Tomoiku Takaku, Toshio Naito

**Affiliations:** ^1^ Department of General Medicine Juntendo University Faculty of Medicine Bunkyo‐Ku Tokyo Japan; ^2^ Department of Hematology Juntendo University School of Medicine Bunkyo‐Ku Tokyo Japan

**Keywords:** COVID‐19 vaccination, subcutaneous panniculitis‐like T‐cell lymphoma, T‐cell lymphoma

## Abstract

This is a case of subcutaneous panniculitis‐like T‐cell lymphoma (SPTCL) was diagnosed by skin biopsy in a patient who presented with fever and erythema nodosum in the umbilicum following mRNA‐1273 COVID‐19 vaccination. COVID‐19 vaccines may cause SPTCL and skin biopsy may help in the diagnosis of erythema nodosum.

## 1 CASE REPORT

Subcutaneous panniculitis‐like T‐cell lymphoma is a rare disease with a relatively good prognosis, although recurrence is frequent. Herein, we report a case of subcutaneous panniculitis‐like T‐cell lymphoma diagnosed in a patient who presented with fever and erythema nodosum after mRNA‐1273 coronavirus disease 2019 vaccination.

**FIGURE 1:**
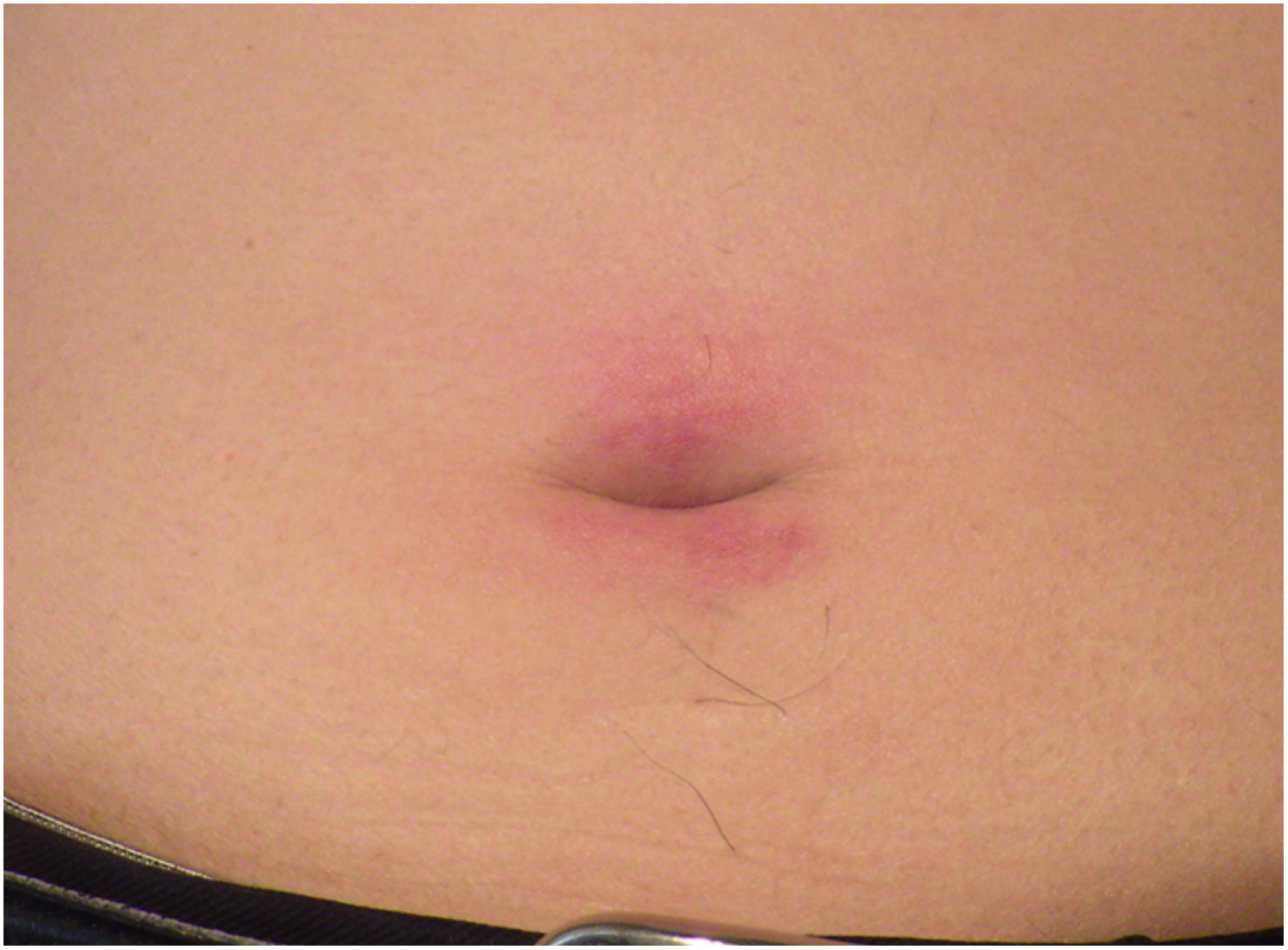
Erythema nodosum around the umbilicus.

**FIGURE 2:**
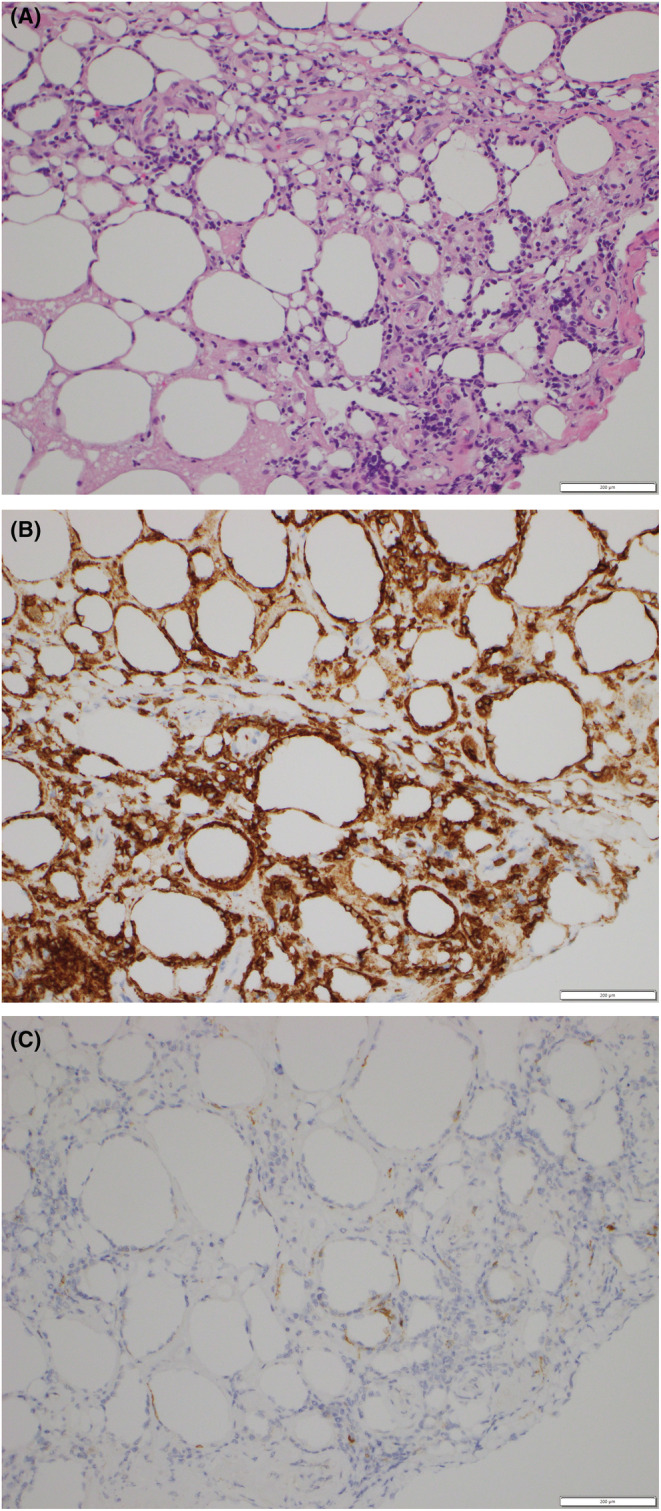
Atypical lymphocytes and associated histiocytes that are rimming the adipocytes, in a lace‐like manner resembling panniculitis. The neoplastic infiltrate was composed of pleomorphic T cells with irregular and hyperchromatic nuclei. Hematoxylin–Eosin stain 400× (A). Immunohistology staining showed positivity for CD8 (B) and CD56 (C).

A 45‐year‐old man with no specific medical history, including cancer, received his third mRNA‐1273 coronavirus disease‐2019 (COVID‐19) vaccination. This was the patient's third vaccination—a booster dose. There were no reactions to the previous vaccinations, both of which were BNT162b2 mRNACOVID‐19, while the third was an mRNA‐1273 COVID‐19 vaccination. Three days later, he developed fever (38.4°C) which lasted for approximately 5 weeks before he arrived at our outpatient clinic. On physical examination, erythema nodosum was observed in the periumbilical region accompanied by pain and warmth. The rash appeared 3 weeks after the COVID‐19 booster vaccination (Figure 1). There was no generalized lymphadenopathy or enlargement of the liver or spleen. Initially, our differential diagnosis included postvaccine fever, bacteremia, periumbilical cellulitis, adult Still's disease, solid tumor metastasis, and pancreatitis. Examinations included upper and lower gastrointestinal endoscopy, contrast‐enhanced CT scan of the trunk, and two sets of blood cultures, none of which showed abnormal findings. Then the skin biopsy was performed to confirm the diagnosis. Histological analysis of the skin biopsy revealed atypical lymphocytes and associated histiocytes that are rimming the adipocytes, in a lace‐like manner resembling panniculitis. The neoplastic infiltrate was composed of pleomorphic T cells with irregular and hyperchromatic nuclei (Figure 2A). Also, it revealed an atypical CD8+ and CD56+ lymphocytic infiltrate in the subcutaneous tissue (Figure 2B,C). The patient was diagnosed with subcutaneous panniculitis‐like T‐cell lymphoma (SPTCL) and treated with a combination of cyclophosphamide, doxorubicin, vincristine, and prednisone. After 3 months of follow‐up, the patient's skin changes and other symptoms had abated.

Subcutaneous panniculitis‐like T‐cell lymphoma (SPTCL) is rare, accounting for 1.3% of all cases of T‐cell lymphomas.^1^ Common symptoms include painless subcutaneous nodules or poorly circumscribed indurated plaques and systemic B symptoms (fever, night sweats, and weight loss) in approximately 60% of patients.^1^ In addition, SPTCL causes hemophagocytic syndrome (HPS) in 13.9% of patients.^2^ Although recurrence is common, the prognosis of this lymphoma subtype is good, with an average 5‐year survival rate of nearly 100%.^2^ Currently, there is no standard treatment for SPTCL, but most cases respond well to systemic corticosteroids or immunosuppressive agents such as etoposide, cyclosporine A, methotrexate, chlorambucil, and bexarotene.^3^ Hematopoietic stem‐cell transplantation is also performed in refractory cases.^3^ There are no specific complications characteristic of SPTCL treatment. There have been no other reports of SPTCL outbreaks following mRNA‐1273 COVID‐19 vaccination. There is only one report of a case of T‐cell lymphoma following the administration of Ad26 viral vector‐based COVID‐19 vaccine (Janssen Pharmaceuticals).^4^ Although the pathogenic mechanism is unclear, it has been suggested that some immune mechanism may be involved in the cause of developing SPTCL after the administration of the COVID‐19 vaccine.^4^ In fact, fever and high inflammatory response may occur after COVID‐19 or the COVID‐19 vaccine due to abnormal activity of innate immunity.^5^ Therefore, the COVID‐19 vaccine may be involved in immune responses. SPTCL has been associated with systemic lupus erythematosus, Sjögren's syndrome, type 1 diabetes, and juvenile idiopathic arthritis and may be affected by an immune response.^1^

Overall, more research is needed to examine possible associations between COVID‐19 vaccination and SPTCL. In this case, COVID‐19 vaccines may cause SPTCL, and skin biopsy at an early stage may help in the diagnosis of erythema nodosum.

## AUTHOR CONTRIBUTIONS

**Sho Ukishima:** Conceptualization; data curation; methodology; writing – original draft; writing – review and editing. **Taiju Miyagami:** Conceptualization; investigation; methodology; project administration; supervision; validation; visualization; writing – original draft; writing – review and editing. **Mari Arikawa:** Conceptualization; formal analysis; methodology; writing – review and editing. **Seiko Kushiro:** Conceptualization; validation; writing – review and editing. **Tomoiku Takaku:** Conceptualization; supervision; validation; writing – original draft; writing – review and editing. **Toshio Naito:** Conceptualization; supervision; validation; writing – review and editing.

## Funding

None.

## CONFLICT OF INTEREST STATEMENT

All authors have no pertinent conflict of interest to report for this manuscript.

## ETHICS STATEMENT

None.

## WRITTEN CONSENT FROM THE PATIENT

Written informed consent was obtained from the patient to publish this report in accordance with the journal's patient consent policy.

## Data Availability

No data were generated in reference to this case report.
